# Diffusion of Minimally Invasive Approach for Lung Cancer Surgery in France: A Nationwide, Population-Based Retrospective Cohort Study

**DOI:** 10.3390/cancers15133283

**Published:** 2023-06-22

**Authors:** Alain Bernard, Jonathan Cottenet, Pierre-Benoit Pages, Catherine Quantin

**Affiliations:** 1 Department of Thoracic and Cardiovascular Surgery, Dijon University Hospital, 21000 Dijon, France; alain.bernard@chu-dijon.fr (A.B.); pierrebenoit.pages@chu-dijon.fr (P.-B.P.); 2 Service de Biostatistiques et d’Information Médicale (DIM), CHU Dijon Bourgogne, Inserm, Université de Bourgogne, CIC 1432, Module Épidémiologie Clinique, 21000 Dijon, France; jonathan.cottenet@chu-dijon.fr; 3 Inserm, High-Dimensional Biostatistics for Drug Safety and Genomics, Le Centre de Recherche en Epidémiologie et Santé des Populations (CESP), Université Paris-Saclay (UVSQ), 94800 Villejuif, France

**Keywords:** lung cancer surgery, minimally invasive approach, variation, region

## Abstract

**Simple Summary:**

The minimally invasive approach (MIA) for lung cancer has gained popularity in recent years thanks to their efficacy and safety. In France, we have no information on the diffusion of MIAs across different hospitals and regions. Our work consisted of evaluating the diffusion of this approach and its variability within each region and from one region to another. Our study showed that the rate of MIAs increased significantly from 2013 to 2020. We also found that the hospital volume, hospital type, and period were significantly related to the adjusted rate of MIAs. The variability between regions was high since 18% of the variance was due to systematic differences between regions. The MIA is now a standard surgical technique used for the treatment of lung cancer in France, even though this technology is mostly used by surgeons practicing in high volume institutions.

**Abstract:**

Background. The minimally invasive approach (MIA) has gained popularity thanks to its efficacy and safety. Our work consisted of evaluating the diffusion of the MIA in hospitals and the variability of this approach (within and between regions). Methods. All patients who underwent limited resection or lobectomy for lung cancer in France were included from the national hospital administrative database (2013–2020). We described between-hospital differences in the MIA rate over four periods (2013–2014, 2015–2016, 2017–2018, and 2019–2020). The potential influence of the hospital volume, hospital type, and period on the adjusted MIA rate was estimated by a multilevel linear regression. Results. From 2013 to 2020, 77,965 patients underwent a lobectomy or limited resection for lung cancer. The rate of the MIA increased significantly over the four periods (50% in 2019–2020). Variability decreased over time in 7/12 regions. The variables included in the multilevel model were significantly related to the adjusted rate of the MIA. Variability between regions was considerable since 18% of the variance was due to systematic differences between regions. Conclusions. We confirm that the MIA is part of the surgical techniques used on a daily basis for the treatment of lung cancer. However, this technology is mostly used by surgeons in high volume institutions.

## 1. Introduction

In France, the incidence of lung cancer (LC) has steadily increased, especially in women (+5% between 2010 and 2018) [1]. LC is the leading cause of cancer-related deaths, with 37,095 deaths recorded in 2020 in France [2]. Surgical resection remains the gold standard for early-stage and locally advanced LC [3]. Several surgical approaches are currently available, including open thoracotomy (OT) and, more recently, minimally invasive techniques such as video-thoracoscopy (VATS) and robot-assisted thoracic surgery (RATS). A survey by the European Society of Thoracic Surgeons (ESTS) reported that senior surgeons performed VATS in less than 5% of their lobectomy cases, while younger consultants reported using VATS in up to 50% of their lobectomy cases [4]. Since then, the minimally invasive approach has gained popularity in recent years, thanks to its efficacy and safety, and is now recommended for early-stage localized LC [3,5,6,7].

In France, the diffusion of the minimally invasive approach among hospitals and within regions has yet to be assessed. Several questions may arise: does the use of this new technology differ according to the type of establishment? Are there differences between regions? This second question underlines the issue of patient access to new technologies according to their place of residence. In order to answer these different questions, we used data extracted from an exhaustive French medico-administrative database.

Our work consisted in evaluating the diffusion of the minimally invasive approach among hospitals and then estimating the distribution of this approach within each French region over the 2013–2020 period. Our secondary objective was to measure the variability of this approach from one region to another.

## 2. Materials and Methods

### 2.1. Data Source and Study Population

All data for patients who underwent pulmonary resection for LC in France from January 2013 to December 2020 were collected from the national hospital administrative database. This database, called PMSI for “Programme de Médicalisation des Systèmes d’Information”, was inspired by the US Medicare system. The reliability and validity of PMSI data have already been assessed [8]. Routinely collected medical information includes the principal diagnosis, secondary diagnoses, and procedures performed. Diagnoses identified during the hospital stay are coded according to the International Classification of Diseases, tenth revision (ICD-10) [8]. We selected patients for whom a diagnosis of primary LC was coded as the principal discharge diagnosis (all C34 codes). Metastatic stages were excluded. Procedures are coded according to the CCAM (Classification Commune des Actes Médicaux). For all patients, LC was confirmed by pathological analyses according to the 2004 World Health Organization classification of LC [8]. Surgery-related variables included the surgical approach (thoracotomy, video-assisted thoracic surgery (VATS), or robot-assisted thoracic surgery (RATS)), the type of resection (limited resection or lobectomy). The surgical approach variable included two classes: open thoracotomy and the minimally invasive approach (including VATS or RATS). Ethics approval for the use of this database was obtained from the French National Commission for Data protection (Commission Nationale de l’Informatique et des Libertés: No 1576793), and this study adhered to the tenets of the Declaration of Helsinki.

### 2.2. Patient Characteristics

Patient age and sex were included as baseline demographic characteristics. From the national administrative database, we included the following comorbidities: pulmonary disease (chronic bronchitis and emphysema), heart disease (coronary artery disease, cardiac arrhythmia, congestive heart failure, valvular heart disease, pulmonary artery hypertension, and pulmonary embolism), peripheral vascular disease, liver disease, cerebrovascular events, neurological diseases (hemiplegia or paraplegia), kidney disease, hematologic disease (leukemia and lymphoma), metabolic disease, anemia, other therapies (preoperative chemotherapy and steroids), and infectious diseases. We also calculated the modified Charlson Comorbidity Index (CCI) as a marker of comorbidity [9].

### 2.3. Region and Hospital Characteristics

Metropolitan France comprises 13 regions: Auvergne-Rhones-Alpes (ARA), Bourgogne-Franche-Comté (BFC), Bretagne (BRE), Centre Val-de-Loire (CVL), Corse (COR), Grand-Est (GE), Hauts-de-France (HdF), Ile-de-France (IdF), Normandie (NOR), Nouvelle Aquitaine (NA), Occitanie (OC), Pays de Loire (PdL), and Provence-Alpes-Côte d’Azur (PACA). For each hospital within a region, we determined the number of times each type of pulmonary resection was performed from 1 January 2013 to 31 December 2020. Hospital volume was defined as the median number of procedures performed per year. For the purpose of the analysis, the hospital volume was represented as a continuous variable that was transformed into a logarithm. Each hospital was categorized as an academic (teaching) hospital, non-academic hospital, non-profit private hospital, or private hospital.

### 2.4. Statistical Analysis

To obtain the adjusted minimally invasive approach rate, we used the hierarchical logistic regression model with “shrinkage” estimators [10]. The adjusted minimally invasive approach rate was determined as the smoothed, adjusted, hospital-specific minimally invasive rate divided by the expected minimally invasive rate. The expected minimally invasive rate was estimated from a fixed-effects component of a hierarchical logistic model regression, using comorbidities, age, sex, modified CCI score, and the type of pulmonary resection as adjustment factors. To estimate the hierarchical logistic regression model, we used a Bayesian method, which seems more flexible and suitable for small sample sizes. As there is no simple closed-form solution for the hierarchical estimator of the adjusted minimally invasive approach rate (and 95% probability interval (PI)), models were estimated using Gibbs sampling. After an initial burn-in of 5000 iterations, parameter estimates were based on the subsequent 5000 draws. Ninety-five percent PIs were obtained through identification of the 2.5th and 97.5th percentiles of the 5000 RAMs. Uncertainty surrounding the estimated adjusted minimally invasive approach rate was quantified by calculating the Bayesian 95% PI.

We separated the time of inclusion into 4 periods: the first, 2013–2014; the second, 2015–2016; the third, 2017–2018; and the fourth, 2019–2020. The Cochran–Armitage test was used to study the evolution of trends over time for patient characteristics. We used several methods to describe between-hospital differences in the minimally invasive approach rate for each of the 4 periods. Different statistics of variation are commonly used: those that describe the median of rates, such as interquartile, interval, or range (IQR) and the extreme and interquartile ratios. The IQR defines the interval or the range between the 1st and the 3rd quartiles of the distribution and measures how the data spreads around the average. The wider the range, the greater the level of dispersion around the mean. The extreme ratio corresponds to the ratio between the maximum and minimum values. The interquartile ratio measured the ratio between the value at the 3rd quartile and this value at the 1st quartile. An extreme or interquartile ratio >2 implies that the maximum value is twice as high as the minimum value or that the value at the 3rd quartile is twice as high as the value at the 1st quartile, respectively.

To estimate the potential influence of different hospital characteristics, such as the hospital volume, type of hospital, and time period, on the adjusted minimally invasive approach rate and after assessing the normality of the variables, we used a multilevel linear regression model with a random intercept for the region. To estimate the variability of the adjusted minimally invasive approach rate between regions, we used the intraclass correlation coefficient (ICC), which was calculated by fitting a multilevel regression model. We then estimated the proportion of variation of this rate between regions and between hospitals of the same region, attributable to hospital characteristics and periods, using two-sequential random effects models. The first model (“empty model”) was estimated with a random region effect without any other variables included in the model. A second random effects model (“full model”) included the hospital characteristics and period. Using standard techniques, we calculated the proportion of variation in the minimally invasive approach rate due to the hospital characteristics and period: change in the variance of the random effect calculated as follows: (variance full model − variance “empty” model)/variance full model.

The calculations for the hierarchical and multilevel logistics regression models were carried out using the STATA 14 software (StataCorp, College Station, TX, USA), and for the full Bayesian analysis, we used the R2jags module (including BUGS) of R version 4.22 software (http://www.r-project.org, accessed on 1 September 2022), so that we could use the BUGS software (Bayesian Inference Using Gibbs Sampling, version 0.60; MRC Biostatistics Unit, Cambridge, UK) via R.

## 3. Results

From January 2013 to 31 December 2020, 77,965 patients underwent a lobectomy or limited resection for LC. Patient characteristics over time are reported in Table 1. The percentage of patients aged 75 years and over increased significantly with time (*p* < 0.0001, Cochran-Armitage test): they represented 18% of the patients operated on for LC in the last period (2019–2020) in France (Table 1). The percentage of women also increased significantly with time (*p* < 0.0001, Cochran-Armitage test): women represented 39% of the operated patients in 2019–2020 (Table 1). The rate of VATS or RATS increased significantly over the four periods (*p* < 0.0001, Cochran-Armitage test), reaching 50% in 2019–2020 (Table 1).

### 3.1. Variations in the Use of the Minimally Invasive Approach in Hospitals over Time

**Figure 1:**
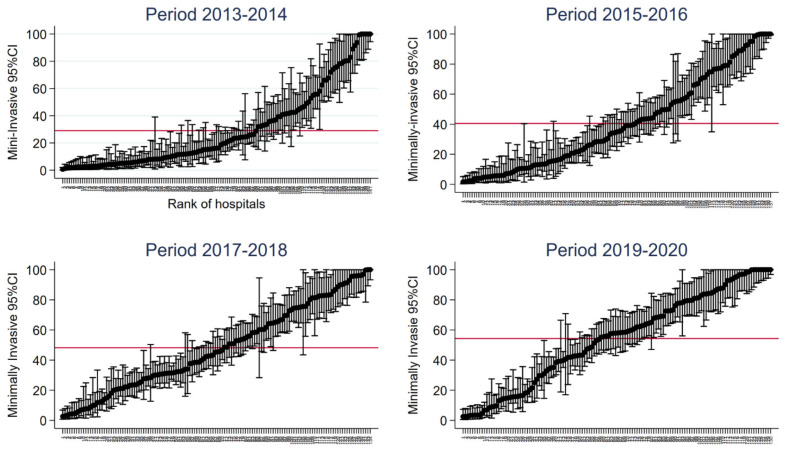
Variation in the rate of minimally invasive procedures among hospitals. (CI, confidence interval.) The red line represents the national rate per period. (X-axis: hospital reference number; Y-axis: adjusted rate of minimally invasive surgery.)

The number of hospitals decreased over the subsequent periods (Table 2). At the same time, the hospital volume dramatically increased (Table 2). All types of hospitals increased their volume of activity over the four periods (Table 2). The median rate for the minimally invasive approach increased over the periods, the increase being higher for academic hospitals and private non-profit hospitals (Table 2). For the 2019–2020 period, the median adjusted rate was 0.68 for academic hospitals and 0.77 for private non-profit hospitals, while it was 0.42 for non-academic and 0.56 for private for-profit hospitals (Table 2). As shown in Figure 1, there was a wide variation in the proportion of minimally invasive techniques between hospitals. The minimally invasive approach was used from 0 to 100% of the interventions depending on the hospital, and this high variation between hospitals remained regardless of the period (Figure 1). The overall mean increased over the four periods as minimally invasive LC surgeries were performed more frequently in hospitals (Figure 1).

### 3.2. Between-Hospital Variations in Adjusted Minimally Invasive Approach Rates in Each Region of France 

The variation in the adjusted rate for the minimally invasive approach between hospitals is reported in Table 3 for each period in each region. The median rate increased over time in the ARA, BRE, HdF, IdF, NA, OC, PACA, and PdL regions (Table 3). The extreme and interquartile ratios are also reported in Table 3. For almost all regions, the estimated variation by extreme or interquartile ratio was high, ranging from 8 to 143 and 2 to 14, respectively, during the first period (2013–2014) (Table 3). According to the extreme ratio, this variability decreased over time for the following regions: BRE, GE, IdF, NOR, OC, PACA, and PdL (Table 3). For the other regions (ARA, BFR, CVL, HdF, and NA), the variability of the minimally invasive rates remained high over the last period 2019–2020, with an extreme ratio ranging from 19 to 36 (Table 3). According to the interquartile ratio, the variability decreased over time for the following regions: ARA, BFR, BRE, HdF, NOR, OC, and PACA (Table 3). For the other regions (CVL, GE, IdF, NA, and PDL), the variability of the minimally invasive rates remained high over the last period 2019–2020, with an interquartile ratio higher than 4 (Table 3).

The minimally invasive approach rate, adjusted for time period, hospital volume per period, and the type of hospital, increased significantly over time (Table 4).

**Figure 2:**
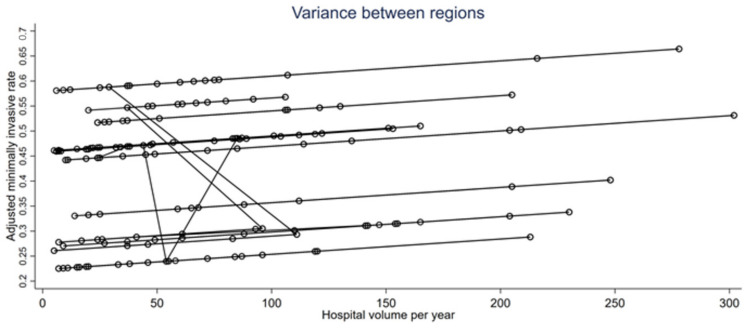
The significant linear relationship between the adjusted minimally invasive approach rate and the hospital volume (defined as the median number of procedures performed per year) of the different hospitals in each region (each circle corresponds to a hospital and each line to a region). We show that not only does the hospital volume have an influence on the spread of minimally invasive methods, but that this trend differs from one region to another, as shown by the space between the curves.

The variability between regions was considerable with an ICC of 0.18, indicating that 18% of the variance was due to systematic differences between regions (Table 4). The three variables included in the multilevel linear regression model (the time period, hospital volume per period, and type of hospital) explained 23% of the variation in the adjusted minimally invasive approach rate within regions and 14% of the variation between regions (Figure 2 and Table 4).

**Figure 3:**
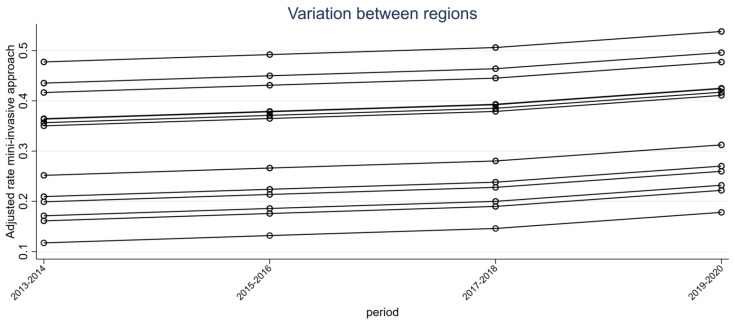
The significant linear relationship between the adjusted minimally invasive approach rate and the time period in each region (each line corresponds to a region). The spread of minimally invasive techniques increases within regions over different periods but differs between regions, as shown by the difference between the curves representing a region.

The variability between regions in the diffusion of minimally invasive surgery in the different periods is confirmed by Figure 3. The implementation of minimally invasive surgery was not homogeneous: some regions were more advanced than others.

## 4. Discussion

In France, the use of the minimally invasive approach for LC surgery has increased steadily in recent years. In 2019–2020, 50% of lobectomies or limited resections were minimally invasive. Intra-regional variations have decreased over time due to the diffusion of this approach, but there are still disparities in practice depending on the type of hospital and the hospital volume. We also found that the diffusion of this technology has not occurred at the same pace in the various French regions. The variability between regions was relatively high considering that 18% of the variance was due to systematic differences between regions, independently of the time period considered.

We showed that the minimally invasive approach was used for 50% of lobectomies or limited resections in 2019–2020 in France. During the same time period in the United States, the rate of minimally invasive procedures was 75.4% [11]. The difference observed with the United States could first be explained by the type of database used. The STS database is not exhaustive for surgical practice, and experienced teams are most likely to contribute to this database. In contrast, all the hospitals in France that perform lung resections for cancer are included in our medical-administrative database. Therefore, as we have shown, hospitals with a low volume perform fewer minimally invasive procedures. The difference in the types of hospitals included in the two databases could therefore explain the differences in the minimally invasive approach rates between the two countries. Indeed, in a study based on the STS database, the authors showed great variability in this rate, ranging from 0% to 100% [8], depending on the center. Our results also confirm the extreme variation between hospitals since the rate of minimally invasive approach varied from 0 to 100%. Moreover, the difference between hospitals remained significant throughout the period.

In our work, we have indeed found that the hospital type and the hospital volume were mainly responsible for the variation between hospitals in the same region. Academic and private non-profit hospitals, which have higher volumes, had higher minimally invasive approach rates of 0.68 and 0.77, respectively, compared to other hospitals. Our work confirms that hospital volume influences the use of the minimally invasive procedure. Minimally invasive techniques require a relatively large learning curve to master [12,13]. Accordingly, surgeons who work in low-volume hospitals will have more difficulty mastering these approaches. We observed that, in France, non-academic and private for-profit hospitals were most likely to have a low volume. These same facilities had a lower minimally invasive approach rate, respectively, 0.42 and 0.56. The difference with respect to the other types of hospitals (academic and private non-profit) was considerable, ranging from 12% to 35%. Not surprisingly, wider regions such as “Hauts-de-France” or “Auvergne-Rhône-Alpes”, which include a large number of hospitals performing LC surgery, showed greater variations. Indeed, these regions have more low-volume hospitals where surgeons tend to prefer open thoracotomy for safety reasons. Our work has also shown that the significant intra-regional variations observed in the first period (2013–2014) have diminished in the fourth period (2019–2020), as technology has become more accessible.

Our study also highlights the high interregional variability (almost 20%) in the rate of minimally invasive approaches, which remained constant regardless of the time period considered. We have thus observed that the spread of this technology, which is potentially beneficial for patients, has not proceeded at the same pace in all French regions. This discrepancy seems regrettable, because our health system should allow all French people to benefit from innovative technologies regardless of their place of residence, especially if these technologies improve the quality of care. The use of minimally invasive techniques is indeed considered an indicator of quality and was recently included in the United States [11]. Several publications have demonstrated that the minimally invasive approach is a safe and feasible technique [6,14,15,16,17]. Some studies have described the benefits of the minimally invasive approach in terms of lymph node dissection, 30-day mortality, and intraoperative conversion rates [6,14,15,16,17]. Three randomized controlled trials have recently been published, demonstrating the quality-of-life benefit of VATS over thoracotomy [18,19,20]. However, there are no randomized controlled trials investigating the potential advantages of RATS over thoracotomy in localized LC. Indeed, currently available data stems from retrospective studies included in meta-analyses or in retrospective studies without a propensity score analysis [16,21]. In terms of postoperative mortality, Zhang’s meta-analysis showed that perioperative mortality was significantly lower among patients who underwent RATS than in patients who underwent thoracotomy (OR 0.14 (95% CI) [0.03–0.59]); *p* = 0.007) [22]. Similarly, Kent et al. found that robot-assisted lobectomies were associated with lower mortality rates compared with thoracotomy [23]. All of these publications were in favor of the minimally invasive approach, leading to it being recommended for early-stage localized LC [3,5].

In our study, we showed to what extent LC surgery practices depend on the hospital type and size. Few studies have assessed national or regional variations in surgical practice [24,25]. One study, concerning congenital heart surgery, showed the disparity in the outcomes for this surgery according to the hospital and, in particular, the hospital volume. The variability was not as marked for hospitals with a high volume [24]. The other study, from an Italian team, was focused on pelvic organ prolapse surgery and, in particular, the place of the approach (laparotomy or laparoscopy or robotic surgery) [25]. This very interesting study reported a wide variation in surgical practices between hospitals [25]. The authors showed that 30.2% of variation for complications could be explained by disparities in individual and hospital characteristics [25]. We believe that a better understanding of these variations is necessary to improve the organization of care and that further studies are needed, whether on lung cancer or other conditions, particularly in the field of oncology. As far as lung cancer surgery is concerned, our work also suggests that it would be useful, in France at least, to consider regionalizing the technical platforms that carry out thoracic procedures to enable surgeons to acquire these minimally invasive techniques more easily, as this would increase the volume of activity per platform.

### Limitations and Strengths

We recognize that our study has some limitations. In particular, for the minimally invasive approaches, which are coded according to the CCAM (Classification Commune des Actes Médicaux), we were not able to distinguish between video-assisted thoracic surgery and robot-assisted surgery since this distinction only appeared in France in mid-2019. Secondly, the results of the present study may be specific to the French healthcare system and thus cannot be generalized to other countries or regions. Another limitation concerns the TNM stage which can influence mortality but cannot be recorded in the PMSI. This is important because it can be assumed that the trends in LC characteristics over time, especially the decrease in tumor size, has allowed surgeons to perform fewer pneumonectomies in recent years. This limitation may be tempered by the fact that metastatic stages were excluded. No details were available in this database concerning the care offer of the facility (the number of beds in the surgical department and intensive care units, the number of days spent in intensive care, and the number of nurses and medical practitioners) or the organization of care (tumor board meetings, the application of recommendations, qualification of the surgeons and years of experience, and surgery performed by residents).

The main strength of our results is related to the large size of our sample (77,965 patients). Our national administrative database, PMSI, is an important tool because it collects the characteristics of patients from all care centers in France. It provides a huge amount of epidemiological information about hospitalized French patients [26,27,28,29,30,31]. Moreover, data pertaining to pulmonary resection for LC are reliable enough to count such patients, as previously shown in other studies [32,33,34,35,36,37].

## 5. Conclusions

In conclusion, this work confirms that the minimally invasive approach has become standard practice for the treatment of lung cancer. We also showed that this technology is mostly used by surgeons practicing in institutions with a high volume. Finally, this study stresses the differences in the use of this technology between regions, showing that our health system does not offer all citizens an equal opportunity to benefit from innovative technologies.

## Figures and Tables

**Table 1:** Annual variation in the characteristics of patients undergoing lung cancer resection.

	Period				
	2013–2014	2015–2016	2017–2018	2019–2020	*p*-Value *
N	16,933	18,778	20,903	21,351	
Age					
38–56	3203 (18.92%)	3397 (18.09%)	3336 (15.96%)	3135 (14.68%)	
57–61	2908 (17.17%)	3029 (16.13%)	3219 (15.40%)	2969 (13.91%)	
62–65	2798 (16.52%)	2972 (15.83%)	3134 (14.99%)	3302 (15.47%)	0.00001
66–69	2850 (16.83%)	3361 (17.90%)	3836 (18.35%)	3898 (18.26%)	
70–74	2461 (14.53%)	2934 (15.62%)	3814 (18.25%)	4222 (19.77%)	
≥75	2713 (16.02%)	3085 (16.43%)	3564 (17.05%)	3825 (17.91%)	
Female	5460 (32.24%)	6528 (34.76%)	7683 (36.76%)	8407 (39.38%)	0.00001
Modified CCI					
0	5829 (34.42%)	6448 (34.34%)	7300 (34.92%)	12,451 (58.32%)	0.00001
1	1762 (10.41%)	1992 (10.61%)	2387 (11.42%)	1750 (8.20%)	
2	1785 (10.54%)	2129 (11.34%)	2533 (12.12%)	1918 (8.98%)	
≥3	7557 (44.63%)	8209 (43.72%)	8683 (41.54%)	5232 (24.50%)	
VATS/RATS	3841 (22.68%)	6489 (34.56%)	9194 (43.98%)	10,734 (50.27%)	0.00001
Limited	3085 (18.22%)	3368 (17.94%)	3673 (17.57%)	3583 (16.78%)	0.0012
Lobectomy	13,848 (81.78%)	15,410 (82.06%)	17,230 (82.43%)	17,768 (83.22%)	

First row has frequencies and second row has column percentages. * Chi^2^ test.

**Table 2:** Annual variation in the hospital characteristics and minimally invasive approach.

	Period			
	2013–2014	2015–2016	2017–2018	2019–2020
Number of hospitals	141	137	134	130
Type of hospitals				
Non academic	31	32	31	30
Academic	27	27	26	26
Private non-profit	13	10	11	9
Private for profit	70	68	66	65
Hospital volume *				
Non academic	48 [30–74]	62 [48–84]	68 [48–114]	77 [55–129]
Academic	183 [147–386]	208 [125–380]	270 [155–443]	284 [161–426]
Private non-profit	125 [35–366]	246 [85–428]	225 [31–464]	258 [188–501]
Private for profit	60 [30–103]	70 [37–124]	93 [43–153]	92 [49–158]
Observed rate of minimally invasive approach **				
Non academic	0.042 [0–0.25]	0.16 [0.04–0.41]	0.23 [0.05–0.61]	0.33 [0.03–0.64]
Academic	0.11 [0.04–0.31]	0.29 [0.13–0.57]	0.46 [0.25–0.66]	0.56 [0.41–0.68]
Private non-profit	0.28 [0.17–0.4]	0.52 [0.38–0.57]	0.6 [0.49–0.77]	0.65 [0.5–0.7]
Private for profit	0.11 [0.05–0.31]	0.23 [0.08- 0.45]	0.34 [0.18–0.55]	0.43 [0.17–0.66]
Overall	0.12 [0.04–0.31]	0.26 [0.08–0.5]	0.37 [0.21–0.61]	0.47 [0.24–0.68]
Adjusted rate of minimally invasive approach **				
Non academic	0.06 [0.04–0.33]	0.22 [0.06–0.55]	0.32 [0.08–0.75]	0.42 [0.07–0.78]
Academic	0.15 [0.06–0.47]	0.42 [0.18–0.7]	0.57 [0.31–0.75]	0.68 [0.53–0.85]
Private non-profit	0.41 [0.24–0.55]	0.65 [0.5–0.79]	0.65 [0.58–0.82]	0.77 [0.63–0.8]
Private for profit	0.16 [0.08–0.42]	0.3 [0.12–0.59]	0.38 [0.23–0.6]	0.56 [0.22–0.8]
Overall	0.12 [0.06–0.43]	0.35 [0.13–0.66]	0.45 [0.25–0.74]	0.58 [0.3–0.8]

*: median of the number of procedures performed by period [interquartile]; **: median [interquartile].

**Table 3:** Between-hospital variation in the adjusted minimally invasive approach rates in each region of France.

	2013–2014		2015–2016		2017–2018		2019–2020	
	Median [IQR]	Extreme Ratio Interquartile Ratio	Median [IQR]	Extreme Ratio Interquartile Ratio	Median [IQR]	Extreme Ratio Interquartile Ratio	Median [IQR]	Extreme Ratio Interquartile Ratio
ARA	0.43	32	0.57	37	0.75	15	0.8	22
	[0.14–0.74]	5.29	[0.35–0.8]	2.29	[0.56–0.9]	1.61	[0.54–1]	1.85
BFR	0.15	15	0.13	15	0.31	6	0.09	19
	[0.05–0.32]	6.40	[0.09–0.35]	3.89	[0.28–0.32]	1.14	[0.08–0.15]	1.88
BRE	0.28	20	0.67	20	0.66	2	0.71	2
	[0.09–0.65]	7.22	[0.27–0.8]	2.96	[0.48–0.83]	1.73	[0.58–0.84]	1.45
CVL	0.1	8	0.194	10	0.31	6	0.3	16
	[0.08–0.2]	2.50	[0.08–0.33]	4.13	[0.3–0.37]	1.23	[0.14–0.56]	4.00
GE	0.075	61	0.302	41	0.24	25	0.39	7
	[0.023–0.21]	9.13	[0.11–0.42]	3.82	[0.12–0.4]	3.33	[0.16–0.64]	4.00
HdF	0.12	37	0.192	37	0.34	27	0.51	36
	[0.04–0.33]	8.25	[0.11–0.35]	3.18	[0.22–0.5]	2.27	[0.38–0.6]	1.58
IdF	0.24	53	0.41	19	0.58	24	0.63	11
	[0.05–0.7]	14.00	[0.13–0.8]	6.15	[0.26–0.84]	3.23	[0.2–0.93]	4.65
NA	0.1	35	0.132	26	0.232	24	0.41	32
	[0.04–0.16]	4.00	[0.06–0.27]	4.50	[0.08–0.33]	4.13	[0.07–0.6]	8.57
NOR	0.21	143	0.435	8	0.46	5	0.59	6
	[0.11–0.6]	5.45	[0.2–0.66]	3.30	[0.32–0.72]	2.25	[0.35–0.9]	2.57
OC	0.24	58	0.51	25	0.462	10	0.67	4
	[0.12–0.41]	3.42	[0.24–0.6]	2.50	[0.33–0.73]	2.21	[0.57–0.9]	1.58
PACA	0.41	33	0.48	6	0.624	4	0.67	3
	[0.15–0.49]	3.27	[0.3–0.85]	2.83	[0.43–0.76]	1.65	[0.53–0.9]	1.70
PdL	0.36	26	0.49	18	0.6	7	0.6	10
	[0.11–0.43]	3.91	[0.24–0.7]	2.92	[0.22–0.78]	3.55	[0.18–0.83]	4.61

Extreme ratio (=maximum/minimum). An interquartile ratio (=3rd quartile/1st quartile) greater than 2 implies that the maximum (the 3rd quartile, respectively) value is twice as high as the minimum (the 1st quartile, respectively) value. IQR: interquartile interval (1st quartile–3rd quartile).

**Table 4:** Multilevel linear regression model.

	Coefficient	*p*-Value	[95% Confidence Interval]	
Period				
2013–2014	0			
2015–2016	0.12	0.001	0.06	0.18
2017–2018	0.18	0.001	0.12	0.24
2019–2020	0.25	0.001	0.19	0.31
Logarithm hospital volume	0.06	0.0001	0.03	0.09
Type of Hospital				
Non-academic	0			
Academic	0.06	0.1	−0.014	0.15
Private non-profit	0.17	0.001	0.07	0.27
Private for-profit	0.03	0.4	−0.035	0.08
Variance between regions	0.015			
Variance within regions	0.067			
Intraclass correlation coefficient	0.18			
Explained variation within regions	0.23			
Explained variation between regions	0.14			

## Data Availability

The use of these data by our department was approved by the National Committee for data protection. We are not allowed to transmit these data. PMSI data are available for researchers who meet the criteria to access these French confidential data (this access is submitted to the approval of the National Committee for data protection) from the national agency for the management of hospitalization (ATIH—Agence technique de l’information sur l’hospitalisation).
